# Roles of Toll-like receptor 2/4, monoacylglycerol lipase, and cyclooxygenase in social defeat stress-induced prostaglandin E_2_ synthesis in the brain and their behavioral relevance

**DOI:** 10.1038/s41598-019-54082-5

**Published:** 2019-11-26

**Authors:** Xiang Nie, Shiho Kitaoka, Masakazu Shinohara, Akira Kakizuka, Shuh Narumiya, Tomoyuki Furuyashiki

**Affiliations:** 1 Division of Pharmacology, Graduate School of Medicine, Kobe University, Hyogo, Japan; 2 Japan Agency for Medical Research and Development (AMED), Tokyo, Japan; 3 Laboratory of Functional Biology, Graduate School of Biostudies, Kyoto University, Kyoto, Japan; 4 Division of Epidemiology, Graduate School of Medicine, Kobe University, Hyogo, Japan; 5 The Integrated Center for Mass Spectrometry, Graduate School of Medicine, Kobe University, Hyogo, Japan; 6 Department of Drug Discovery Medicine, Graduate School of Medicine, Kyoto University, Kyoto, Japan

**Keywords:** Neuroimmunology, Stress and resilience

## Abstract

Inflammation in the brain and periphery has been associated with stress-related pathology of mental illness. We have shown that prostaglandin (PG) E_2_, an arachidonic acid-derived lipid mediator, and innate immune receptors Toll-like receptor (TLR) 2/4 are crucial for repeated stress-induced behavioral changes in rodents. However, how the stress induces PGE_2_ synthesis in the brain and whether TLR2/4 are involved in the PGE_2_ synthesis remain unknown. Using mice lacking TLR2 and TLR4 in combination, here we show that social defeat stress (SDS) induced the PGE_2_ synthesis in subcortical, but not cortical, tissues in a TLR2/4-dependent manner. It is known that PGE_2_ in the brain is mainly derived by monoacylglycerol lipase (MAGL)-mediated conversion of endocannabinoid 2-arachidonoylglycerol to free-arachidonic acid, a substrate for cyclooxygenase (COX) for PGE_2_ synthesis. We found that TLR2/4 deletion reduced the mRNA expression of MAGL and COX1 in subcortical tissues after repeated SDS. Perturbation of MAGL and COX1 as well as COX2 abolished SDS-induced PGE_2_ synthesis in subcortical tissues. Furthermore, systemic administration of JZL184, an MAGL inhibitor, abolished repeated SDS-induced social avoidance. These results suggest that SDS induces PGE_2_ synthesis in subcortical regions of the brain via the MAGL-COX pathway in a TLR2/4-dependent manner, thereby leading to social avoidance.

## Introduction

Stress is a strain of physical and psychological functions caused by aversive and demanding events. Whereas stress is thought to induce adaptive biological responses to promote survival and well-being, prolonged or excessive stress may cause depression and elevated anxiety as well as cognitive impairments, and can be a risk factor for mental illnesses^1–3^. To understand the mechanism underlying stress-induced behavioral disturbances, various animal models of repeated stress, such as chronic mild stress and repeated social defeat stress (SDS), have been used^1,2^. Previous studies using these stress models have shown that repeated stress alters neuronal structures and functions in multiple brain areas along with behavioral disturbances^3^.

Clinical studies have reported altered levels of inflammation-related molecules including prostaglandin (PG) E_2_ in blood samples of depressive patients^4–8^. PGE_2_ is a bioactive lipid derived from free arachidonic acid by sequential actions of cyclooxygenase (COX) and PGE synthase, and binds to its receptors, EP1, EP2, EP3 and EP4, to exert various physiological and pathophysiological actions^9^. Notably, meta-analyses have shown that treatment with non-steroidal anti-inflammatory drugs (NSAIDs), especially celecoxib, that inhibit PG synthesis has therapeutic effects in depressive patients^10–12^. These observations suggest a crucial role of PGE_2_ in the pathology of depression. In parallel, rodent studies using repeated stress models have shown the involvement of PGE_2_ in repeated stress-induced behavioral changes^13,14^. For example, our previous study indicated that repeated exposure to SDS attenuates dopaminergic response in the medial prefrontal cortex through PGE receptor EP1, thereby leading to social avoidance^13^. Consistently, repeated SDS increases the PGE_2_ content in subcortical tissues of the brains^13^. Furthermore, genetic deletion or pharmacological inhibition of COX1 abolishes repeated SDS-induced social avoidance^13^. These findings suggest that the COX1-PGE_2_-EP1 pathway is crucial for repeated SDS-induced behavioral changes. However, how SDS induces the PGE_2_ synthesis in the brain remains elusive.

Previous studies using immune cells including microglia have shown that cytosolic phospholipase A_2_ liberates arachidonic acid from the sn-2 position of membrane phospholipid for COX-dependent PGE_2_ production^15^. By contrast, it has recently been shown that a major pool, if not all, of free arachidonic acid for PG production in several organs including the brain is derived from the hydrolysis of endocannabinoid 2-arachinodonoylglycerol (2AG) by monoacylglycerol lipase (MAGL)^16^. Thus, which enzymatic pathway is used for SDS-induced PGE_2_ production in the brain remains to be determined.

Previous studies have shown that lipopolysaccharide (LPS), a bacterial endotoxin that activates an innate immune receptor Toll-like receptor (TLR) 4, induces PGE_2_ synthesis in cultured microglia^17–20^. TLRs are composed of several isoforms, each of which recognizes specific molecular patterns of microbes including LPS^21^. It has recently been suggested that endogenous TLR ligands are released from the cells upon cellular damage and induce inflammation and tissue remodeling without apparent infection^22,23^. Our recent study has demonstrated a crucial role of TLR2 and TLR4 (TLR2/4) for repeated SDS-induced social avoidance and elevated anxiety^24^. Furthermore, TLR2/4 mediate repeated SDS-induced microglial activation in the medial prefrontal cortex, which underlies concomitant neuronal and behavioral changes^24^. Thus, whether and how TLR2/4 are involved in SDS-induced PGE_2_ synthesis in the brain needs to be investigated.

Using mice lacking TLR2 and TLR4 in combination, here we show a crucial role of TLR2/4 in SDS-induced PGE_2_ synthesis in the subcortical regions of the brain. We also used JZL184, SC-560 and SC-236 that are inhibitors for MAGL, COX1 and COX2, respectively, and obtained the findings suggesting that the MAGL-COX pathway is responsible for SDS-induced subcortical PGE_2_ synthesis and social avoidance.

## Methods

### Animals

*Tlr2/4* double knockout (TLR-DKO) mice in a C57BL/6N background were purchased from Oriental Bio Service. *Ptgs1* (i.e. the gene encoding COX1) knockout mice (COX1-KO) in a C57BL/6N were purchased from Taconic. To make all the mice congenic to the C57BL/6N strain, these mice were backcrossed with C57BL/6N mice for more than 10 generations. Adult male C57BL/6N mice and male ICR mice retired from breeding were purchased from Japan SLC and CLEA Japan, respectively. After arrival, mice were housed in a group of four mice in a specific pathogen-free and temperature- and humidity-controlled vivarium under a 12-h light, 12-h dark cycle (light on between 0800 and 2000 or between 0600 and 1800) with free access to chow and water. All procedures for animal care and use were in accordance with the National Institutes of Health Guide for the Care and Use of Laboratory Animals and were approved by the Animal Care and Use Committees of Kyoto University Graduate School of Medicine and Kobe University Graduate School of Medicine.

### Social defeat stress

Single and repeated exposure to SDS was applied as described previously with minor modifications^24^. Briefly, male ICR mice were screened based on their aggressiveness to a male C57BL/6N mouse, as measured by the latency and the number of attacks during the observation period (180 s), and were used as aggressor mice for SDS. Before SDS, 7∼12-week-old male mice were isolated with free access to chow and water for 1 week. Each of the isolated mice to be defeated (i.e. intruder mice) was introduced and kept in the home cage of a resident aggressor ICR mouse for 10 min daily for 1 day (single SDS) or 10 consecutive days (repeated SDS). After the 10 min defeat episode, the mice were returned to their home cages and kept isolated until SDS on the next day. The pairs of defeated and aggressor mice were randomized daily to minimize the variability in the aggressiveness of aggressor mice. SDS was applied between 1600 and 1900 h in a sound-attenuated room in dim light. Naïve mice, which did not receive SDS, were placed in a novel cage for 10 min daily (i.e. cage transfer) for 1 day or 10 consecutive days as a control to compare with those which receive single or repeated SDS, respectively. We included all the data for the analyses without any exclusion. After repeated SDS, the social interaction test and the elevated plus maze test were performed. These behavioral tests were performed as previously described^24^.

### Measurement of PGE_2_ and IL-1β contents using EIA

**Figure 1:**
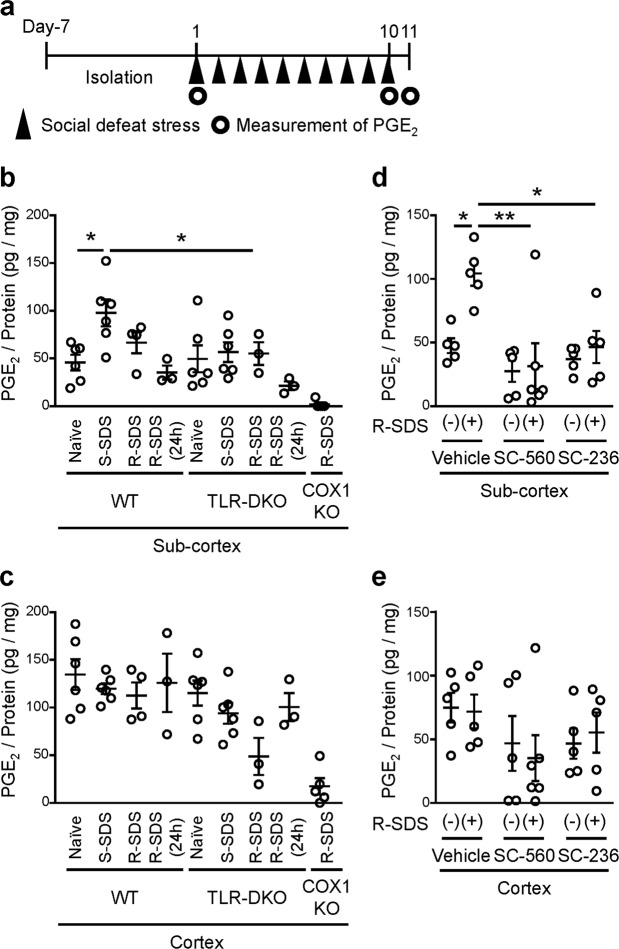
TLR2/4 are involved in COX-dependent PGE_2_ synthesis in subcortical tissues. (**a**) Schedule of experiments in this study. After social isolation for 1 week, a male wild-type or TLR-DKO mouse was subjected to SDS through an encounter with an ICR aggressor mouse for 10 min daily for 1 day (Day 1) or 10 consecutive days (Day 1 through Day 10). The PGE_2_ measurement was performed immediately after the SDS exposure on Day 1 or Day 10, or at 24 h after the last SDS exposure (i.e. on Day 11). (**b,c**) The PGE_2_ contents in subcortical (**b**) and cortical (**c**) tissues of wild-type (WT), TLR-DKO and COX1-KO mice without SDS (naïve), immediately after single SDS (S-SDS), and immediately and at 24 h after the last SDS session of repeated SDS (R-SDS). (**d,e**) The PGE_2_ contents in subcortical (**d**) and cortical (**e**) tissues of wild-type mice administrated with SC-560 and SC-236 that are COX1 and COX2 inhibitors, respectively, as well as vehicle without SDS and immediately after the last session of repeated SDS (R-SDS). *P < 0.05, **P < 0.01 for Bonferroni’s multiple comparisons test. Data are shown as means ± SEM.

**Figure 2:**
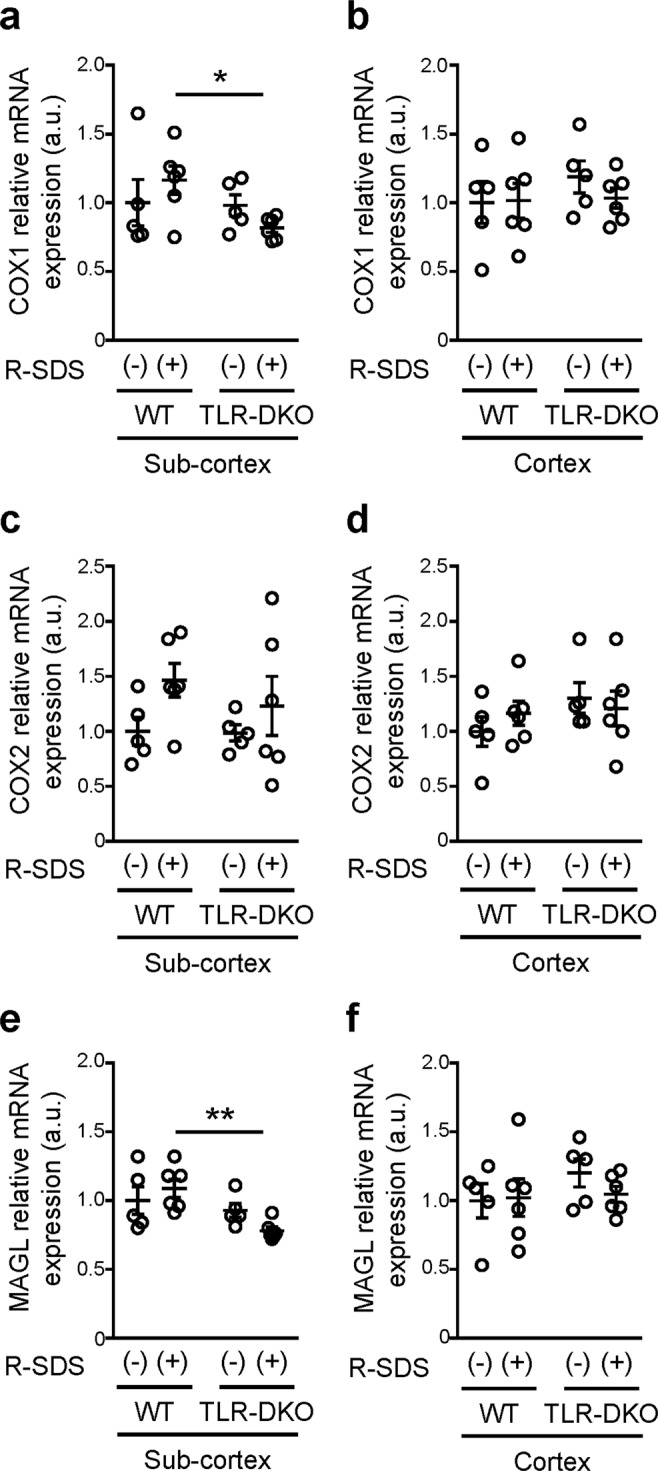
TLR2/4 are involved in the expression of COX1 and MAGL after repeated social defeat stress in subcortical tissues. The relative mRNA expression levels of COX1 (**a**,**b**), COX2 (**c,d**) and MAGL (**e,f**) in subcortical (**a,c,e**) and cortical (**b,d,f**) tissues of wild-type (WT) and TLR2/4 double knockout (TLR-DKO) mice with or without repeated SDS (R-SDS). *P < 0.05, **P < 0.01 for Bonferroni’s multiple comparisons test. Data are shown as means ± SEM.

Measurement of PGE_2_ contents in brain homogenates by enzyme immunoassay (EIA) was performed as described previously with minor modifications^13^. Briefly, a mouse was decapitated immediately after SDS or cage transfer, except that the decapitation was performed 24 h after the last session of repeated SDS in Fig. 1. A brain was removed from the mouse and placed in ice-cold Dulbecco’s modified phosphate-buffered saline (D-PBS) with 25 μM indomethacin to prevent artificial PGE_2_ production during brain processing. The brain was cut at the coronal plane between the olfactory bulb and the cerebral cortex and at the coronal plane between the midbrain and the cerebellum, and the brain tissue between these coronal planes was used for the measurements. In Figs. 1 and 2, the cortical tissue containing the cerebral cortex and the hippocampus and the remaining brain tissue (i.e. subcortical tissue) were separated. All these procedures were completed within 30 s after decapitation. The brain tissues were snap-frozen in liquid nitrogen and kept at −80 °C until use. For EIA, the brain tissues were homogenized in the homogenization buffer (0.1 M sodium phosphate, pH 7.4, containing 1 mM EDTA and 25 μM indomethacin) using a Polytron homogenizer (Kinematica) or Micro Smash (Tomy). The homogenized solution was centrifuged at 20,000 × g for 15 min, and the supernatants were subjected to PGE_2_ EIA (Cayman Chemicals) according to the manufacturer’s protocol. The precipitations after the centrifugation were resuspended and sonicated in RIPA buffer (25 mM Tris-HCl, pH 7.6, 150 mM NaCl, 1.0% NP-40, 1.0% sodium deoxycholate, 0.1% SDS), and their protein concentrations were determined using BCA assay (Fujifilm Wako Pure Chemical) according to the manufacturer’s protocol. For the measurement of IL-1β contents in brain homogenates, the same supernatant obtained for PGE_2_ EIA was subjected to IL-1β EIA (eBioscience). The contents of PGE_2_ and IL-1β were normalized to the protein concentrations.

### Measurement of PGE_2_ and free arachidonic acid contents using liquid chromatography-tandem mass spectrometry

Measurement of PGE_2_ and free arachidonic acid contents by liquid chromatography-tandem mass spectrometry (LC-MS/MS) was performed as previously described with minor modifications^25^. Briefly, a mouse was decapitated immediately after the last exposure to repeated SDS or cage transfer. The brain tissues were homogenized in 100% methanol using Micro Smash. The homogenized solution was centrifuged at 15,000 × g for 5 min at 4 °C, and the supernatants were added with 500 pg of d_4_-prostaglandin E_2_ and d_8_-5-hydroxyeicosatetraenoic acid (5-HETE) as deuterated internal standards. Then the samples were subjected to solid-phase extraction on C18 columns, and the extracted lipids were detected with a QTRAP 6500 instrument (SCIEX) equipped with an LC-30AD high-performance LC system (Shimadzu). Quantification was performed on the basis of peak areas on the multiple reaction monitoring chromatograph, with linear calibration curves being obtained with authentic standards for each compound.

### Quantitative RT-PCR

The cortical and subcortical tissues obtained from the same mice used for PGE_2_ measurements were used for quantitative RT-PCR. Quantitative RT-PCR was performed as previously described^24^. Briefly, total RNA was extracted from these tissues using NucleoSpin RNA/Protein Kit (Macherey-Nagel) according to the manufacturer’s protocol. The resultant RNA was used to obtain cDNA using a High Capacity cDNA Reverse Transcription Kit (Life Technologies). All PCR experiments were conducted in duplicate using SYBR Premix Ex Taq (Takara Bio), and fluorescent SYBR Green signals were automatically detected and analyzed with a CFX384 Touch Real-Time PCR Detection System (Bio-Rad). Primers for PCR are as follows: for mouse 18S ribosomal RNA (18S rRNA), 5′-GTA ACC CGT TGA ACC CCA TT-3′ and 5′-CCA TCC AAT CGG TAG TAG CG-3′; for mouse COX1, 5′-CCT CTT TCC AGG AGC TCA CA-3′ and 5′-TCG ATG TCA CCG TAC AGC TC-3′; for mouse COX2, 5′-GGG AGT CTG GAA CAT TGT GAA-3′ and 5′-TGT CAA TCA AAT ATG ATC TGG ATG T-3′; for mouse MAGL, 5′-GGA ACA AGT CGG AGG TTG AC-3′ and 5′-GCA GCT GTA TGC CAA AGC AC-3′. The values were normalized to those of 18S rRNA in the same cDNA samples. The levels were then normalized to those of the control groups.

### Drugs

JZL184, an inhibitor for MAGL (Cayman Chemical), was administered to C57BL/6 N mice, as described previously with minor modifications^16^. To test its effects on the PGE_2_ contents, JZL184 dissolved in 18:1:1 mixture of saline, ethanol and Cremophor (Nacalai) or vehicle solution without JZL184 was injected intraperitoneally (i.p.) at 40 mg/kg at least 60 min before single exposure to SDS. To test its effects on repeated SDS-induced behavioral changes, JZL184 was administrated 6 h before each exposure to SDS.

SC-560 (Merck) and SC-236 (Cayman Chemical) that are inhibitors for COX1 and COX2, respectively, were administered to C57BL/6N mice, as described previously with minor modifications^13^. SC-560 and SC-236 dissolved in DMSO or vehicle solution alone were injected i.p. at 5 mg/kg at 30 min before the last exposure to SDS (i.e. 10th SDS).

### Statistical analyses

Data are shown as means ± SEM. For comparison between two groups, unpaired Student’s *t* test was used. For comparison of more than two groups, multiple comparison tests with Bonferroni’s correction were used. The analyses were performed with PRISM 7.0 software (GraphPad). *P* values less than 0.05 are considered to be significant.

## Results

### TLR2/4 are crucial for social defeat stress-induced PGE_2_ synthesis in the brain

**Figure 3:**
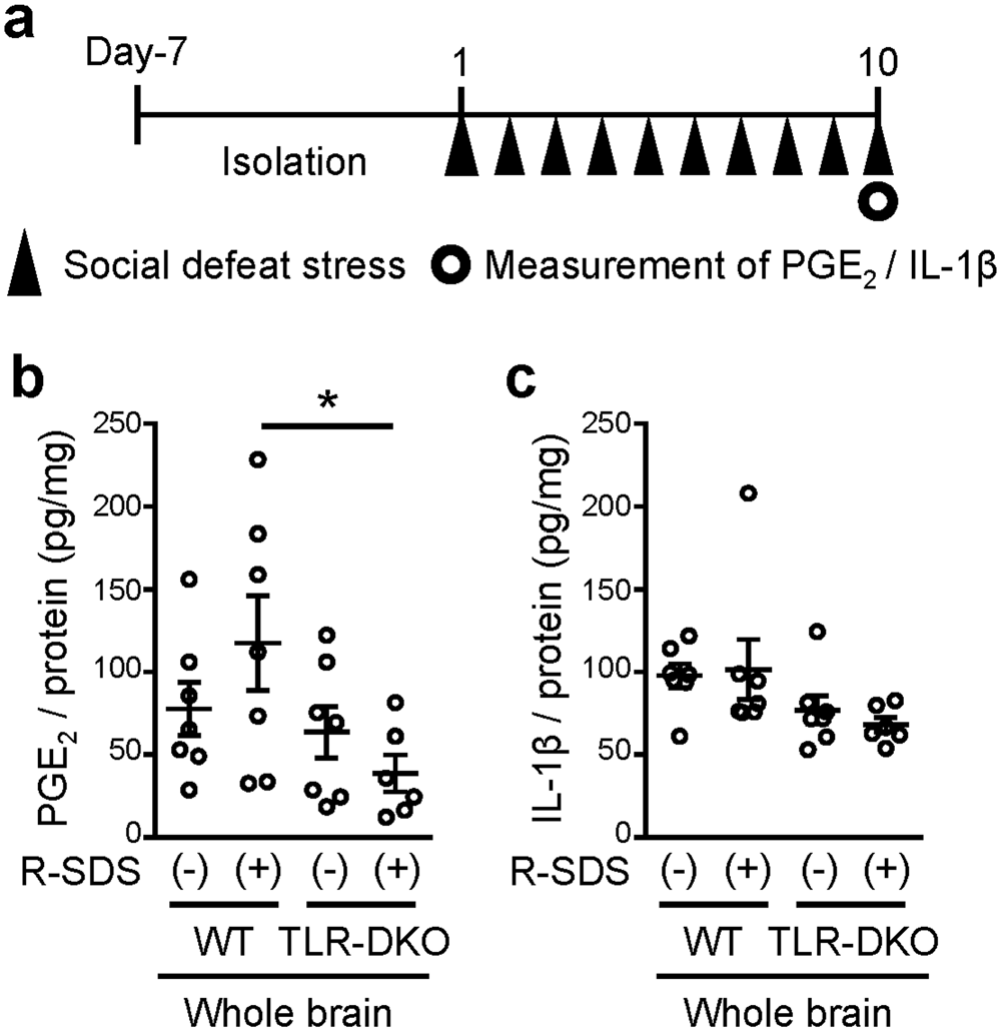
TLR2/4 are crucial for repeated social defeat stress-induced PGE_2_ synthesis in the brain. (**a**) Schedule of experiments in this study. After social isolation for 1 week, a male wild-type or TLR-DKO mouse was subjected to SDS through an encounter with an ICR aggressor mouse for 10 min daily for 10 consecutive days (Day 10). The measurement of PGE_2_ and IL-1β was performed immediately after the last SDS exposure on Day 10. (**b,c**) The contents of PGE_2_ (**b**) and IL-1β (**c**) in whole brains of wild-type (WT) and TLR2/4 double knockout (TLR-DKO) mice treated with or without repeated SDS (R-SDS). The contents of PGE_2_ and IL-1β were normalized to the protein concentrations. *P < 0.05 for Bonferroni’s multiple comparisons test. Data are shown as means ± SEM.

To examine whether TLR2/4 are involved in SDS-induced PGE_2_ synthesis in the brain, we subjected wild-type mice and TLR-DKO mice to repeated SDS (Fig. 3a). An adult male mouse of each genotype (i.e. intruder mouse) was transferred to the home cage of an aggressor ICR mouse for 10 min daily for 10 consecutive days (Fig. 3a). The intruder mouse was immediately attacked by the ICR mouse, and both wild-type and TLR-DKO mice took a submissive posture, a behavioral sign of SDS, in which a mouse stood upright with the abdomen exposed to the ICR mouse, as previously reported^24^. The brains of the defeated mice were collected immediately after the last SDS session and subjected to PGE_2_ measurement. SDS increased the PGE_2_ content in the whole brain, and this increase was significantly reduced in TLR-DKO mice (Fig. 3b). This finding shows a crucial role of TLR2/4 in SDS-induced PGE_2_ synthesis in the brain. Although we mainly used EIA to measure the PGE_2_ contents in this study, we confirmed that EIA and LC-MS/MS yield consistent results regarding SDS-induced increase in the PGE_2_ contents in the brain (see Fig. 1b,c and Supplementary Fig. 1). Since IL-1β has been suggested to be involved in chronic stress-induced depressive-like behavior, we also measured the IL-1β content in the same brain homogenates as those used for the PGE_2_ measurement. However, since the brains were collected immediately after the last SDS session, neither SDS nor TLR2/4 deficiency altered the IL-1β content in the brain (Fig. 3c).

Since we previously reported that SDS-induced inflammatory responses in the medial prefrontal cortex are augmented with repetition of stress, we next compared the PGE_2_ synthesis upon single vs repeated SDS (Fig. 1a). To examine the PGE_2_ synthesis between cortical and subcortical regions, we also divided the brains of defeated mice into cortical tissues including the cerebral cortex and the hippocampus and subcortical tissues including the midbrain for the PGE_2_ measurement. Single exposure to SDS increased the PGE_2_ content in subcortical tissues (Fig. 1b). This PGE_2_ increase appeared to be attenuated with repetition of SDS (Fig. 1b), although repeated SDS still significantly increased the PGE_2_ contents in subcortical tissues (Fig. 1d). TLR2/4 deficiency abolished SDS-induced PGE_2_ synthesis in subcortical tissues (Fig. 1b). By contrast, neither single nor repeated SDS altered the PGE_2_ content in cortical tissues of wild-type mice (Fig. 1c). These findings indicate that SDS induces TLR2/4-dependent PGE_2_ synthesis specifically in subcortical regions. It was noted that the decrease in the PGE_2_ contents in TLR-DKO mice was not associated with an increase in the contents of free arachidonic acid, a precursor for PGE_2_ synthesis (Supplementary Fig. 1), suggesting that TLR2/4 may be involved in the supply of free arachidonic acid after repeated SDS.

### TLR2/4 is involved in the expression of monoacylglycerol lipase and cyclooxygenase-1 in subcortical tissues after repeated social defeat stress

We previously reported that COX1 is crucial for repeated SDS-induced social avoidance^13^. It has also been shown that MAGL hydrolyzes 2AG to generate a major pool of free arachidonic acid as a precursor for PG synthesis in the brain^16^. Thus, we examined whether TLR2/4 affect the mRNA expression of MAGL, COX1 and COX2 with or without repeated SDS. TLR2/4 deficiency reduced the expression of MAGL and COX1 in subcortical tissues, but not in cortical tissues, after repeated SDS (Fig. 2). This reduction was not observed without SDS. The expression of COX2 was not significantly affected by TLR2/4 deficiency. These findings suggest that TLR2/4 are involved in the expression of MAGL and COX1 for SDS-induced PGE_2_ synthesis in subcortical regions.

### Monoacylglycerol lipase and cyclooxygenases are crucial for social defeat stress-induced PGE_2_ synthesis in the brain

**Figure 4:**
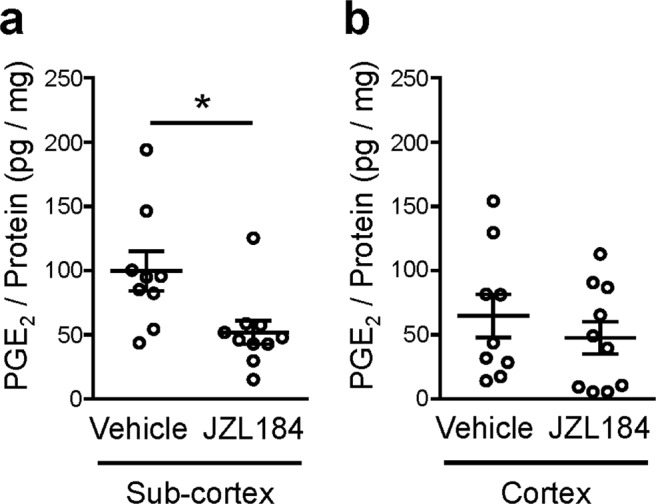
JZL184, an inhibitor for MAGL, suppresses social defeat stress-induced PGE_2_ synthesis in subcortical tissues. The PGE_2_ contents in subcortical (**a**) and cortical (**b**) tissues of wild-type mice treated with vehicle or JZL184 (40 mg/kg, i.p.) at least 60 min prior to single SDS. Brains were obtained immediately after single SDS and subjected to the PGE_2_ measurement. *P < 0.05 for unpaired *t* test. Da*t*a are shown as means ± SEM.

Since TLR2/4 are involved in the COX1 expression after repeated SDS, we examined the PGE_2_ contents in subcortical and cortical tissues of COX1-KO mice. In COX1-KO mice, the PGE_2_ content after repeated SDS was reduced to negligible levels in both subcortical and cortical tissues (Fig. 1b,c), indicating the role of COX1 in SDS-induced PGE_2_ synthesis. To test the roles of COX1 as well as COX2 at the exact time of SDS exposure, we administered SC-560 and SC-236 that are inhibitors for COX1 and COX2, respectively, immediately before the last exposure to repeated SDS, and measured the PGE_2_ contents immediately after that. Both inhibitors abolished SDS-induced increase in the PGE_2_ contents in subcortical tissues (Fig. 1d,e), suggesting the involvement of both COX1 and COX2. By contrast, the PGE_2_ contents in cortical tissues were not significantly affected. We also examined whether MAGL is involved in SDS-induced PGE_2_ synthesis in the brain. JZL184, an MAGL selective inhibitor, was intraperitoneally administered to mice at least 60 min prior to SDS. Treatment with JZL184 decreased the PGE_2_ content immediately after SDS in subcortical, but not cortical, tissues (Fig. 4). Collectively, these findings indicate that MAGL and COX are crucial for SDS-induced PGE_2_ synthesis in subcortical regions.

### Inhibition of monoacylglycerol lipase abolishes repeated social defeat stress-induced social avoidance

**Figure 5:**
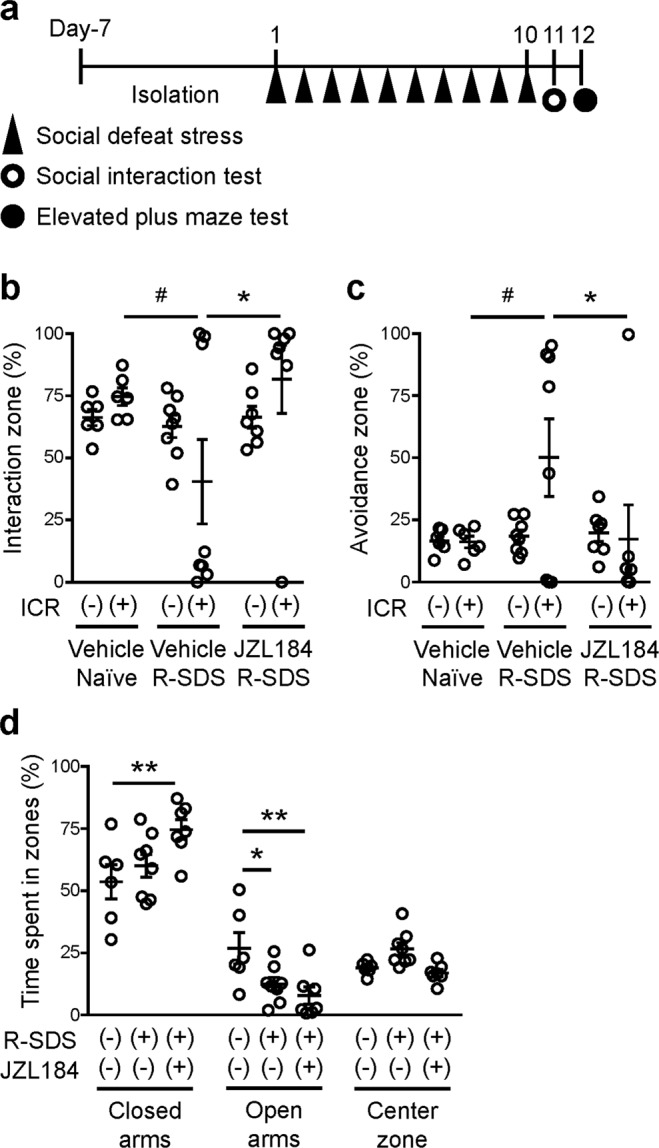
JZL184, an inhibitor for MAGL, suppresses social defeat stress-induced social avoidance. (**a**) Schedule of experiments. After social isolation for 1 week, a male wild-type mouse was subjected to SDS through an encounter with an ICR aggressor mouse for 10 min daily for 10 consecutive days (Day 1 through Day 10). JZL184 or vehicle was administered at 6 h before each SDS exposure. The social interaction test and the elevated anxiety test were performed after repeated SDS to analyze repeated SDS-induced social avoidance and elevated anxiety, respectively. (**b,c**) The effect of JZL184 on repeated SDS-induced social avoidance. The durations in the interaction (**b**) or avoidance (**c**) zone without or with an ICR mouse during the social interaction test were measured for wild-type mice without SDS (naïve) or with repeated SDS (R-SDS) which received i.p. injection of either vehicle or JZL184. (**d**) The effect of JZL184 on repeated SDS-induced elevated anxiety. The proportions of the time spent in the closed arms, open arms and center zone in the elevated plus maze test were measured for the same mice analyzed for the social interaction test. ^#^*P* < 0.1, *P < 0.05, **P < 0.01 for Bonferroni’s multiple comparison test. Data are shown as means ± SEM.

Finally, we examined whether MAGL-mediated PGE_2_ synthesis is involved in repeated SDS-induced behavioral changes. We systemically administered JZL184, a selective inhibitor for MAGL, or its vehicle throughout the course of repeated SDS (Fig. 5a), and found that the JZL184 administrations abolished repeated SDS-induced social avoidance (Fig. 5b,c). By contrast, the elevated anxiety induced by repeated SDS was spared (Fig. 5d). These findings suggest that MAGL-mediated PGE_2_ synthesis is involved in a subset of repeated SDS-induced behavioral changes.

## Discussion

Whereas PGE_2_ signaling is crucial for repeated SDS-induced behavioral changes, how SDS increases the PGE_2_ synthesis in the brain remains unknown. In the present study, SDS increased the PGE_2_ content in subcortical, but not cortical, tissues in a TLR2/4-dependent manner. Our findings indicate that MAGL and COX mediate SDS-induced PGE_2_ synthesis in subcortical tissues, and that TLR2/4 is involved in their expression after repeated SDS. Furthermore, pharmacological inhibition of MAGL abolished repeated SDS-induced social avoidance. These findings suggest that the MAGL-COX pathway that is upregulated by TLR2/4 mediates SDS-induced PGE_2_ synthesis in subcortical regions for the induction of social avoidance (Supplementary Fig. 2).

Since MAGL is crucial for SDS-induced PGE_2_ synthesis in subcortical tissues, the synthesis of 2AG could be involved. Synaptic/neuronal activity induces 2AG synthesis at postsynaptic sites^26^, so that the site of 2AG synthesis could determine a brain region in which PGE_2_ is synthesized. PGE_2_ attenuates the dopaminergic pathway to the medial prefrontal cortex via EP1 receptor for repeated SDS-induced social avoidance^13^. We reported that EP1 receptor is located at the GABAergic synapses that are formed on midbrain dopamine neurons and augments inhibitory inputs to these neurons^27^. Thus, 2AG synthesized from midbrain dopamine neurons or other neurons in the vicinity could be used for MAGL-mediated PGE_2_ synthesis. It should be noted that MAGL inhibition could also increase the 2AG level and thus CB1 activity, which may contribute to the suppression of repeated SDS-induced social avoidance together with PGE_2_. Indeed, stressful stimuli induce 2AG synthesis in subcortical regions, such as the amygdala, and attenuates stress-induced behavioral changes via CB1 receptor^28,29^. The balance between 2AG and PGE_2_, which is regulated by MAGL activity, could determine stress susceptibility. Whereas our findings favor a role of MAGL in SDS-induced PGE_2_ synthesis, we cannot exclude a possible role of cytosolic phospholipase A_2_, which warrants future investigation.

MAGL expression has been reported in various cell types in the brain including neurons, astrocytes and microglia^30,31^. MAGL in neurons and astrocytes has been shown to be responsible for 2AG hydrolysis in the brain without or with LPS stimulation^30^. Since COX1 expression is enriched in microglia in the brain^13^, free arachidonic acid derived from 2AG could be transported from neurons and/or astrocytes to microglia for COX1-mediated PGE_2_ synthesis. Free arachidonic acid has been shown to be shuttled between different cell types at least in culture^30^. Alternatively, since COX2 is expressed in amygdala neurons^32^, COX2 could utilize free arachidonic acid derived from MAGL in the same neurons for the PGE_2_ synthesis in subcortical regions. Cell type-specific deletion of MAGL and COX is useful to address which cell types are involved in the MAGL-COX pathway upon SDS exposure.

Since both SC-560 and SC-236 abolished SDS-induced PGE_2_ synthesis in subcortical tissues, both COX1 and COX2 appear to be involved. Which COX isoform directly mediates this PGE_2_ synthesis remains to be determined. By contrast, we reported that SC-560, but not SC-236, at the same doses abolished repeated SDS-induced social avoidance^13^. Thus, PGE_2_ synthesis could cooperate with other bioactive lipids derived from COX1 for the behavioral change. A lipidomics approach is useful for comprehensive analysis of bioactive lipids involved in repeated SDS-induced behavioral changes.

Our findings indicate a role of TLR2/4 in the MAGL-COX pathway for SDS-induced PGE_2_ synthesis. Consistently, free arachidonic acid derived from 2AG by MAGL is used as a precursor for the PGE_2_ synthesis in the brain induced by LPS, a TLR4 agonist^16^. Previous studies using animal models of chronic inflammation have identified several candidates for endogenous TLR2/4 ligands^24,33,34^. Since single and repeated SDS increased PGE_2_ synthesis immediately in subcortical tissues, endogenous TLR2/4 ligand(s) released upon SDS could immediately stimulate PGE_2_ synthesis. In addition, TLR2/4 are involved in the mRNA expression of MAGL and COX1 in subcortical regions after repeated SDS. Thus, TLR2/4-induced transcriptional activation could also increase MAGL-mediated hydrolysis of 2AG to free arachidonic acid and subsequent COX1-mediated PGE_2_ synthesis.

In conclusion, the present study showed crucial roles of TLR2/4, MAGL and COX in SDS-induced PGE_2_ synthesis and their behavioral relevance using knockout mice and selective inhibitors. To elucidate roles and actions of these molecules in SDS-induced neural and behavioral changes paves the way for understanding the stress-related pathology of mental illnesses and promoting therapeutic development for mental illnesses.

## Supplementary information

Supplementary information

## Data Availability

The datasets generated and/or analyzed during the present study are available from the corresponding author upon reasonable request.
