# Supplementary material for: Roles of Toll-like receptor 2/4, monoacylglycerol lipase, and cyclooxygenase in social defeat stress-induced prostaglandin E2 synthesis in the brain and their behavioral relevance
Source: Sci Rep. 2019 Nov 26;9:17548. doi: 10.1038/s41598-019-54082-5 (PMC6879743; doi:10.1038/s41598-019-54082-5)
Supplement: Supplementary file 1 — Supplementary information [file 41598_2019_54082_MOESM1_ESM.pdf]

Supplementary Information

Roles of Toll-like receptor 2/4, monoacylglycerol lipase, and cyclooxygenase in social defeat stress-induced prostaglandin E_2 synthesis in the brain and their behavioral relevance

[1-3]Xiang Nie, [1,2]Shiho Kitaoka, [4]Masakazu Shinohara, [3]Akira Kakizuka, [5]Shuh Narumiya and [1,2]Tomoyuki Furuyashiki

[1]Division of Pharmacology, Graduate School of Medicine, Kobe University, Hyogo, Japan
[2]Japan Agency for Medical Research and Development (AMED), Tokyo, Japan
[3]Laboratory of Functional Biology, Graduate School of Biostudies, Kyoto University, Kyoto, Japan
[4]Division of Epidemiology, Graduate School of Medicine, Kobe University, Hyogo, Japan
[5]Department of Drug Discovery Medicine, Graduate School of Medicine, Kyoto University, Kyoto, Japan

Correspondence should be addressed to Tomoyuki Furuyashiki
7-5-1 Kusunoki-cho, Chuo-ku, Kobe, Hyogo 650-0017, Japan
TEL +81-78-382-5440; FAX +81-78-382-5459
tfuruya@med.kobe-u.ac.jp

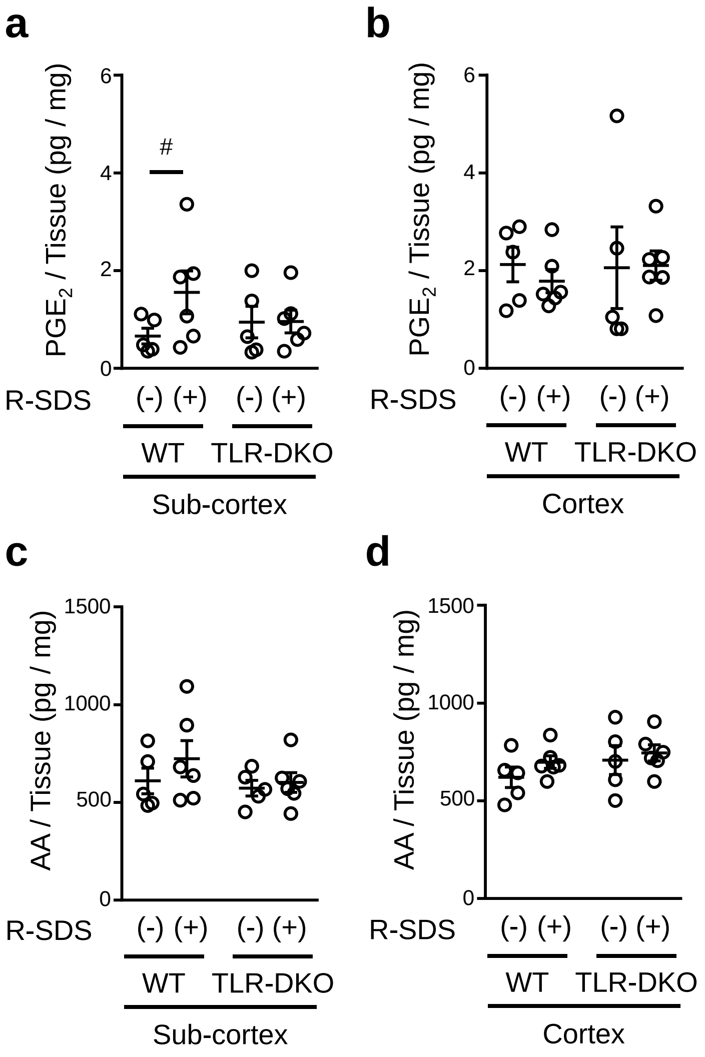

Supplementary Figure S1. LC-MS/MS confirmed that social defeat stress increases the PGE_2 contents in subcortical tissues in a TLR2/4-dependent manner. The contents of PGE_2 (a, b) and free arachidonic acid (AA) (c, d) in subcortical (a, c) and cortical (b, d) tissues of wild-type (WT) and TLR2/4 double knockout (TLR-DKO) mice with or without repeated SDS (R-SDS). #P = 0.09 for unpaired t test. Data are shown as means ± SEM.

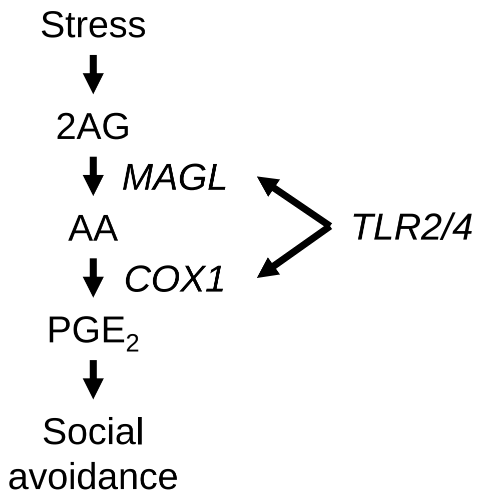

Supplementary Figure S2. Our proposal about stress-induced PGE_2 synthesis in the brain and its behavioral significance based on this study. Our findings suggest that stress increases the PGE_2 synthesis in subcortical regions of the brain through enzymatic activities of MAGL and COX1, the expression of which is enhanced by TLR2/4 activity, thereby leading to social avoidance. 2AG, 2-arachidonoylglycerol; AA, arachidonic acid; COX1, cyclooxygenase-1; MAGL, monoacylglycerol lipase; PGE_2, prostaglandin E_2; TLR, Toll-like receptor.
